# Landscape and environmental influences on *Mycobacterium ulcerans* distribution among aquatic sites in Ghana

**DOI:** 10.1371/journal.pone.0176375

**Published:** 2017-04-24

**Authors:** Shannon M. Pileggi, Heather Jordan, Julie A. Clennon, Ellen Whitney, M. Eric Benbow, Richard Merritt, Mollie McIntosh, Ryan Kimbirauskas, Pamela Small, Daniel Boakye, Charles Quaye, Jiaguo Qi, Lindsay Campbell, Jenni Gronseth, Edwin Ampadu, William Opare, Lance A. Waller

**Affiliations:** 1 Department of Statistics, California Polytechnic State University, San Luis Obispo, California, United States of America; 2 Department of Biological Sciences, Mississippi State University, Starkville, Mississippi, United States of America; 3 Department of Biostatistics and Bioinformatics, Rollins School of Public Health, Emory University, Atlanta, Georgia, United States of America; 4 International Association of National Public Health Institutes’ Office, Emory University, Atlanta, Georgia, United States of America; 5 Department of Entomology, Michigan State University, East Lansing, Michigan, United States of America; 6 Department of Osteopathic Medical Specialties, Michigan State University, East Lansing, Michigan, United States of America; 7 Department of Biology, Xavier University, Cincinnati, Ohio, United States of America; 8 Department of Microbiology, University of Tennessee, Knoxville, Tennessee, United States of America; 9 Parasitology Department, Noguchi Memorial Institute for Medical Research, University of Ghana, Accra, Ghana; 10 Center for Global Change and Earth Observations, Michigan State University, East Lansing, Michigan, United States of America; 11 Department of Ecology and Evolutionary Biology, University of Kansas, Lawrence, Kansas, United States of America; 12 National Buruli ulcer Control Programme, Accra, Ghana; University of Minnesota, UNITED STATES

## Abstract

Buruli ulcer, caused by *Mycobacterium ulcerans*, is highly endemic in West Africa. While the mode of transmission is unknown, many studies associate Buruli ulcer with different types of water exposure. We present results from the largest study to date to test for *M*. *ulcerans* in aquatic sites and identify environmental attributes associated with its presence. Environmental samples from 98 aquatic sites in the Greater Accra, Ashanti, and Volta regions of Ghana were tested for the presence of *M*. *ulcerans* DNA by polymerase chain reaction. The proportion of aquatic sites positive for *M*. *ulcerans* varied by region: Ashanti 66% (N = 39), Greater Accra 34% (N = 29), and Volta 0% (N = 30). We explored the spatial distribution of *M*. *ulcerans* positive and negative water bodies and found no significant clusters. We also determined both highly localized water attributes and broad scale remotely sensed land cover and terrain environmental characteristics associated with *M*. *ulcerans* presence through logistic regression. Our results concur with published results regarding conditions suitable for *M*. *ulcerans* growth and associations with Buruli ulcer disease burden with regards to water characteristics and disturbed environments, but differ from others with regards to spatial associations and topographic effects such as elevation and wetness. While our results suggest *M*. *ulcerans* is an environmental organism existing in a specific ecological niche, they also reveal variation in the elements defining this niche across the sites considered. In addition, despite the causal association between Buruli ulcer and *M*. *ulcerans*, we observed no significant statistical association between case reports of Buruli ulcer and presence of *M*. *ulcerans* in nearby waterbodies.

## Introduction

Buruli ulcer (BU) is a neglected tropical disease (NTD) caused by infection with *Mycobacterium ulcerans*. This potentially debilitating skin disease often begins as a painless nodule and if left untreated can ulcerate, resulting in permanent scarring and disability [1]. Details of clinical symptoms, diagnosis, and treatment are reviewed elsewhere [1–3]. BU cases have been reported in at least 31 countries spread across Africa, Asia, Australia, and Latin America, demonstrating increasing prevalence and expanding geographic distribution during the past century [3]. Endemic in areas of sub-Saharan Africa, countries in West Africa including Benin, Côte d'Ivoire, and Ghana have the highest burden of the disease [2]. Accurate surveillance data reflecting the true disease incidence in West Africa remains elusive due to local variations in aspects such as case confirmation, access to care, diagnosis, and reporting practices.

Unfortunately, much research on Buruli ulcer is still quite speculative, and the best treatment strategy is early case detection rather than disease prevention. In order to target disease prevention efforts, we need to understand the epidemiology of the disease, the transmission cycle, and the habitat of the disease causing pathogen *M*. *ulcerans*. Advanced knowledge of these items has led to comprehensive disease control strategies for other NTDs that include some combination of mass drug administration to endemic populations, vector control through pesticide spraying, biological control though introduction of new species to replace vectors, alterations to the environment, and clean water and sanitation. In light of the many transmission-centered and epidemiological studies on Buruli ulcer, we present the largest study to date on the ecological and geographical habitat surrounding *M*. *ulcerans* in hope of providing insight to this under-studied component of Buruli ulcer disease.

Epidemiological studies have found BU incidence to be associated with water exposure through swimming, domestic water-related activities, and proximity to water [1, 4–12]. Furthermore, many studies suggest that BU is associated with disturbed environments, such as deforested areas and farmlands [9, 13–18]. An environmental pathogen with a distribution in nature thought to be greater than that of the disease, *M*. *ulcerans* has been detected in both endemic and non-endemic sites [19, 20]. Studies have verified the presence of *M*. *ulcerans* DNA in many areas of aquatic systems including suspended material in water, detritus, biofilm, and aquatic insects [8, 19–27]. *M*. *ulcerans* DNA has also been found among many aquatic invertebrates collected from 27 aquatic sites of both endemic and non-endemic communities of Ghana [8, 19]. Although many vectors and reservoirs of the disease have been hypothesized, the mode of transmission has not been conclusively identified. Some hypothesized transmission models include mosquitoes or aquatic insects as a vector, aerosol transmission, or contact through an open skin lesion [6, 8, 9, 22, 23, 28–33]. An extensive review of the ecology and transmission of BU is provided by Merritt et al. (2010) [32, 34].

Studies undertaken in Ghana, Côte d’Ivoire, Cameroon, and Benin have examined the geographical patterns of BU disease endemic areas taking into account landscape and environmental factors [14, 15, 17, 18, 35–37]. Positive associations with BU incidence were found with mean arsenic content of soil, proximity to gold mining sites, irrigated rice crops area, agriculture, forest, potential maximum soil water retention, and wetness index variability. Negative associations with BU were found with dam surface area, urban land cover, and mean elevation. One study also reported geographic clusters of communities with higher than expected and lower than expected disease prevalence, as well as evidence of spatial structure in the geographic distribution of BU cases [15, 17].

Although these studies of BU incidence and prevalence have provided important information for understanding geographic and environmental associations with human disease, similar studies evaluating the factors driving pathogen distribution in the environment have not been conducted. This study sought to investigate the spatial distribution of *M*. *ulcerans* among aquatic sites in Ghana and identify environmental characteristics associated with the presence of *M*. *ulcerans*. We hypothesized that the presence of *M*. *ulcerans* was associated with both broad scale environmental features as well as highly localized characteristics of aquatic systems. At the most localized level, we measured several physical and chemical properties of the aquatic systems themselves. For broad scale environmental features, we used remotely sensed observations to infer landscape and land use/land cover properties among the same aquatic systems. Finally, as an initial test of local similarity between the geographic distribution of the pathogen and disease, we assessed the association of *M*. *ulcerans* presence and the Buruli ulcer disease reporting history of the local community.

## Methods

### Study area

**Fig 1:**
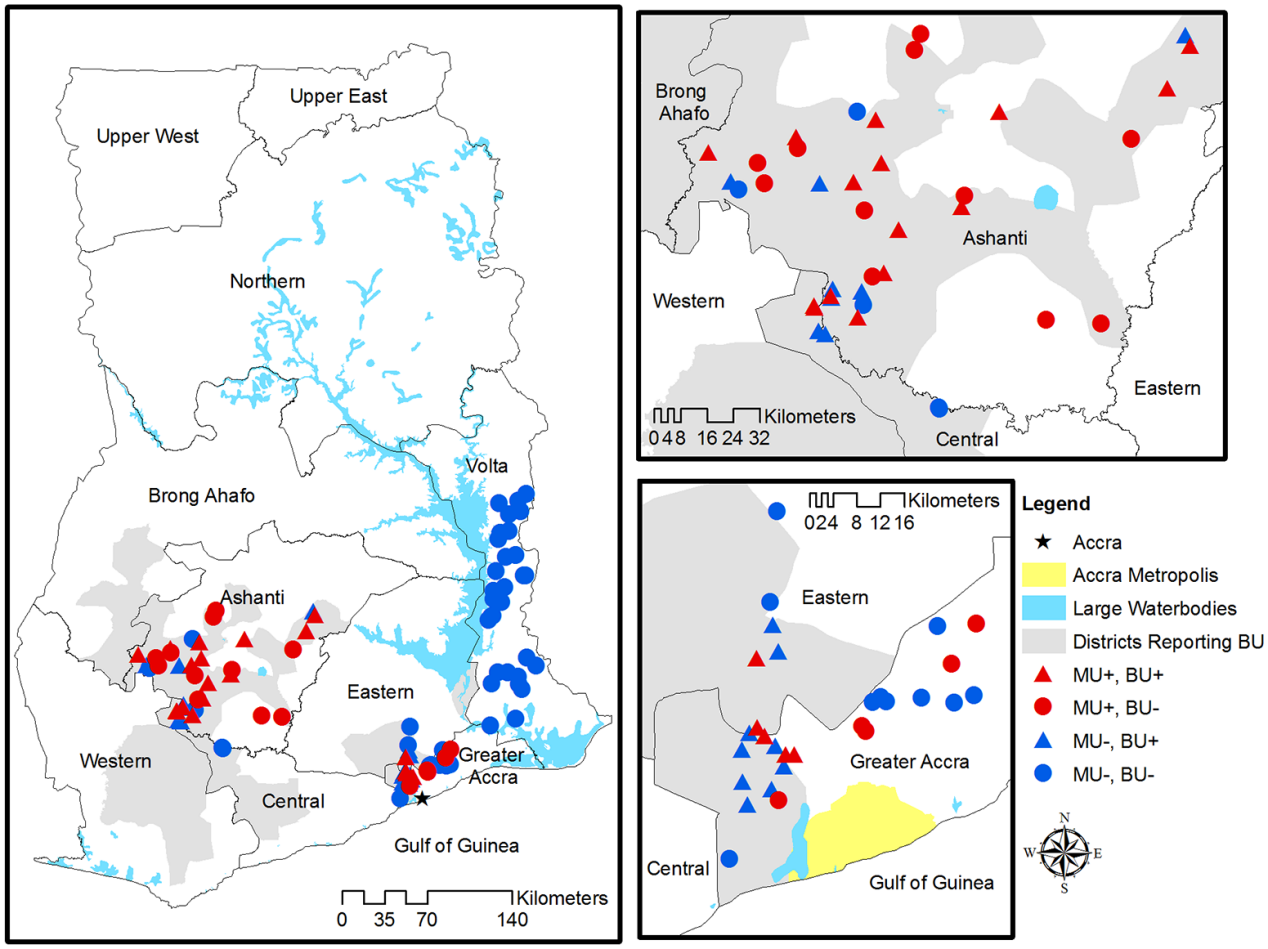
Locations of sampled aquatic sites in the Ashanti, Greater Accra, and Volta regions of Ghana. Administrative districts that reported cases from 2004–07 are shaded in gray. Red symbols indicate *M*. *ulcerans* positive sites, whereas blue symbols indicate *M*. *ulcerans* negative sites. Triangles represent sites that reported cases 2004–07, and circles represent sites with no reported cases 2004–07.

Cases of Buruli ulcer are reported in six out of ten regions in Ghana, with varying levels of frequency. We focused on three geographic areas representing a highly endemic region (Ashanti—the majority of districts report cases of BU), a mixed endemic region (Greater Accra—some districts report cases of BU), and a non-endemic region (Volta—no districts report cases BU). It was of particular interest to establish whether or not *M*. *ulcerans* could be detected in the Volta region where no cases are reported as this had not been studied on a large scale to date. Due to ease of access and lack of clear district borders on the ground, some of the sampled districts did not actually fall within the proper regional borders. A total of 98 sites were sampled in five regions of Ghana: Greater Accra (N = 24), Eastern (N = 5), Ashanti (N = 34), Central (N = 5), and Volta (N = 30) (Fig 1). By reason of geographic proximity, the sites sampled in the Eastern and Central regions of Ghana are henceforth classified with the Greater Accra and Ashanti sites, respectively. Representatives from the National Buruli ulcer Control Programme obtained verbal consent from local village chiefs to collect samples. There were no vertebrates used in this study and no endangered species were encountered or otherwise affected by the study.

Within each site, sampled water bodies were selected based on discussions with community leaders with regards to daily and frequent domestic water use; all water bodies were located within or near (<200m) the villages. Aquatic environmental sampling was performed from 2005–2007 during the dry season between late June to early August of each year and took place on a single date in each village. Different aquatic habitats were sampled to include streams, rivers, wetlands, ponds, and reservoirs, and were classified as lentic (still) or lotic (flowing).

### Buruli ulcer surveillance records

The BU surveillance data of the sampled communities were obtained from the Ghana Health Services, National Buruli ulcer Control Programme. As validation surveys have found BU cases in communities where no case reporting occurred (P. C. Small, Whitney et al., unpublished data), it is possible (indeed probable) that at least some non-reporting communities have cases. Therefore, we consider disease reporting history at two levels. *Community level reporting* is defined as the presence of reported BU cases in the sampled communities from 2004–2007. In contrast, *district level reporting* is defined based on whether the sampled community is located within a district that reported BU cases in the same years (Fig 1). Gray shaded areas indicate districts reporting BU, whereas the color of the points (red vs blue) indicates whether or not the community reported BU cases.

### Sample collection and detection of *M*. *ulcerans*

Briefly, DNA was isolated from environmental matrices that included water filtrand and macrophytes using a protocol adapted from Lamour and Finley [38]. Additionally, *M*. *ulcerans* and *M*. *marinum* strains, and nuclease-free water were used as positive and negative controls, respectively. Positive and negative controls were included with each extraction. DNA was stored at −20°C until further use. PCR was carried out using methods described by McIntosh et al. (2014) [26], Williamson et al. (2008) [19], and Williamson et al. (2014) [39] where all samples were initially subjected to amplification of IS*2404*. Positive samples were further assayed for the presence of the enoyl reductase (ER) domain encoded on the plasmid responsible for mycolactone production, as previously described [19, 26, 39–41]. Samples found to be ER positive were profiled when possible using variable number tandem repeat profile targeting a number of loci [19, 26, 39]. Standard operating procedures for quality assurance of molecular analyses were strictly followed according to the Quality Assurance/Quality Control Guidance for Laboratories performing PCR analyses on Environmental Samples and microbial source tracking by the Environmental Protection Agency, USA [42]. If any of the environmental samples contained *M*. *ulcerans* DNA then the corresponding aquatic site was identified as *M*. *ulcerans* positive.

### Water characteristics

A variety of physical and chemical properties were evaluated for each aquatic site. One-liter water samples were collected to evaluate physicochemical characteristics. Several parameters (e.g., dissolved oxygen, temperature, conductivity, suspended solids, pH) were measured *in situ* using a YSI 6600 Data Sonde (Yellow Springs Instruments, Inc., OH). Water samples taken to measure other water chemistry variables were immediately stored on ice and then frozen until analysis at the Environmental Chemistry Division of the Water Research Institute, Ghana using established and standard water quality methods [19].

### Remotely sensed covariates

We utilized an existing land use / land cover (LULC) classification published in Wagner et al. (2008) that was derived from dry season 2000 and 2002 Landsat EMT+ 30 m resolution satellite imagery [15]. Raw images were obtained from the University of Maryland Global Land Cover Facility (http://glcf.umiacs.umd.edu/), geometrically corrected, and then projected to UTM Zone 30 N [43]. An unsupervised classification with 100 initial classes on the principle axis with pseudo-color for 10 iterations or 95% convergence was run in Erdas Imagine, reducing the number of initial classes from 100 to 22 [15]. These 22 classes were further reduced to six classes, eliminating categories with more than one land cover type and aggregating subclasses to more generalized classes, such as evergreen forest to forest. The final classification consisted of six general land cover classes, including cropland, forest, shrubland, urban, water, and wetlands. Classified land cover was masked within circular buffers surrounding sampled sites at distances of 0.1, 0.5, 1.0, and 5.0 km, with the shortest 0.1 km buffer providing a 3 x 3 window of neighboring classified pixels surrounding the buffer centroid.

A digital elevation model (DEM) was derived from NASA Shuttle Radar Topographic Mission (SRTM) (2000) data with a 3 arc second (90m) resolution at a WRS-2 unfilled finished A processing level, obtained from the University of Maryland Global Land cover Facility (http://glcf.umiacs.umd.edu/data/srtm). The DEM gaps were filled and compound topographic index, or wetness index, was calculated using the following equation [44]:

$$WI=\text{ln}\left(\frac{\left(FA+1.0\right)\times 90m}{slope+0.0001}\right)$$

The minimum, maximum, mean, and standard deviation of the wetness index for buffer diameter sizes of 0.5, 1.0, and 5 km were also calculated as an approximate measure of potential land surface moisture content and its spatial variability. Lastly, site-specific values of elevation were extracted. All environmental covariates were calculated and extracted in ArcGIS 9.3.1 (ESRI Inc., Redlands, CA).

Data are available from the Dryad Digital Repository: http://dx.doi.org/10.5061/dryad.br47r [45].

### Statistical analysis

Ripley’s *K* function [46] was used to test for the presence and scale of any patterns of spatial clustering of *M*. *ulcerans* positive sites relative to negative sites using the *splancs* package in R (http://www.r-project.org/) [47]. Geographic regions were assessed separately for clustering patterns, and the distances at which clustering patterns were evaluated were approximately one-third of the distances separating the two furthest sites in each region. We calculated the differences between the *K* functions that summarized the spatial distribution of *M*. *ulcerans* positive and negative sites (case-control *K* function) and assessed significance via Monte Carlo simulation (999).

To investigate location and significance of individual clusters of *M*. *ulcerans* positive sites, we calculated Kulldorff's Bernoulli spatial scan statistic using circular windows for the Ashanti and Accra areas separately [48]. The statistical significance of potential clusters was evaluated through Monte Carlo hypothesis testing (999 simulations) in SaTScan 8.0 (www.satscan.org) [49] at the 0.05 significance level.

Logistic regression was used to identify variables associated with the presence of *M*. *ulcerans* among the aquatic sites using SAS software version 9.2 (SAS Institute Inc., Cary, NC). Most of the physicochemical water characteristics were log transformed or plus one log transformed due to skewed distributions. The land use / land cover (LULC) covariates considered for model selection included the percent of pixels characterized by an LULC type within the specified buffer distance, as well as by a presence/absence indicator corresponding to specific LULC types within the buffer. Model selection was based on Akaike’s Information Criterion (AIC), an information-theoretic approach for selecting a best model from a set of candidate models [50]. AIC was adjusted for small sample size (AICc) because of the low ratio of the sample size to the number of parameters. The model with the lowest AICc was regarded as the best fitting model of those considered. Due to the high degree of correlation among LULC variables as well as water variables, a set of candidate variables were identified for model building. Only one LULC covariate at each buffer distance which resulted in the greatest reduction of AICc was considered in model selection, and only uncorrelated water quality covariates (r<0.3) with the greatest reduction in AICc were considered in model selection. Region interactions were considered with each candidate covariate. We focused on five best-fitting models constructed using five sets of candidate covariates, namely: (1) water, (2) LULC, (3) DEM (terrain), (4) LULC+DEM (landscape), (5) all sets of covariates (overall).

Model fit was assessed by a variety of methods for the final model constructed from all sets of covariates. Standardized residuals were used to check for the presence of outliers, and observations were evaluated to identify those with high leverage [51]. In addition, the residuals from the Greater Accra and Ashanti regions were assessed separately for spatial autocorrelation by the empirical semivariogram using the *geoR* package in R. The semivariogram estimated variability between distinct pairs of sites as a function of the distance *h* between them. Simulation envelopes (n = 999) were constructed by Monte Carlo simulation in order to test the null hypothesis of spatial independence among the residuals [52].

In addition to assessments of associations between *M*. *ulcerans* presence and landscape variables, we also explored the association between reported BU cases and *M*. *ulcerans* presence. The unadjusted association was tested by Pearson’s chi-square test at the 0.05 significance level. The association adjusted for environmental covariates was assessed by entering the BU reporting history variables individually into the best fitting logistic regression model. If the updated model’s AICc was within 2 units than that of the best fitting model then the BU reporting history variable could be deemed competitive with the best fitting model; if the AICc of the updated model decreased by more than 2 units from the AICc of the best fitting model then the updated model was considered improved in model fit [50].

## Results

Though no cases of BU had been reported from the Volta region during our study period, we sought to determine if this was due to the absence of *M*. *ulcerans* in the Volta region. Indeed, environmental sampling did not detect *M*. *ulcerans* in this region (N = 30). Therefore, for the present study, data from the Volta region were excluded from further analyses in order to investigate factors relating to variation in *M*. *ulcerans* presence.

A higher proportion of aquatic sites tested positive for *M*. *ulcerans* in the Ashanti sites (66%, N = 39) than in the Greater Accra region (34%, N = 29) (Table 1). Physical and chemical properties of water from sites varied by region (Table 1, S1, S2 and S3 Figs). Ashanti sites were at a higher elevation than Accra sites, which were located closer to the coast (median elevation for Accra = 46 meters above sea level, Ashanti = 175 m). Both Accra and Ashanti had similar at-site median wetness index, however the average wetness index within buffers around the sites was greater in Accra than Ashanti. Both regions exhibit similar average wetness index variability within buffers around the site.

**Table 1:** Descriptive statistics of site characteristics in the Greater Accra, Ashanti and Volta regions. Summary statistics are presented as n (%) or median (min, max).

Characteristic	Greater Accra (N = 29)	Ashanti (N = 39)	Volta (N = 30)
**General**			
*M*. *ulcerans* present	10 (34%)	26 (67%)	0 (0%)
Community level reporting	14 (48%)	23 (59%)	0 (0%)
District level reporting	20 (69%)	37 (95%)	0 (0%)
Lentic aquatic system (still)	22 (76%)	13 (33%)	14 (47%)
Elevation	46.0 (15.0, 152.0)	175.0 (100.0, 378.0)	130.5 (2.0, 262.0)
**Water Variables**			
Calcium (mg/L)	17.6 (0, 92.2)	9.6 (1.6, 22.4)	7.2 (0, 23.2)
Calcium hardness as CaCO_3_ (mg/l)	44.1 (0, 231)	24 (1.2, 56.1)	18 (0, 58.1)
Carbon trioxide (mg/L)	109 (1.2, 449)	41.5 (12.2, 151)	50 (14.6, 142)
Chlorine (mg/L)	26.8 (1, 645)	7.9 (1, 67.5)	5.5 (0, 55.6)
Chlorophyll (mg/L)	11 (4.5, 76.1)	8.7 (0.3, 125.7)	5.7 (1.1, 43.1)
Color apparent (Hz)	35 (1.2, 500)	30 (5, 180)	36.5 (2.5, 110)
Dissolved oxygen percent saturation	35 (0.2, 134.8)	56.6 (0.3, 90.3)	54.8 (4.7, 98.2)
Field specific conductivity (μS/cm)	5.6 (4.2, 7.9)	4.8 (3.7, 6.5)	4.6 (3.6, 6)
Field temperature (Celcius)	26.5 (24.3, 32)	24.2 (23.2, 27.8)	25.2 (23.6, 29.3)
Field turbidity (NTU)	17.1 (0.1, 331.5)	34.4 (0, 353.7)	34.3 (1.8, 140.2)
Iron (mg/L)	1.2 (0, 7.2)	2.5 (0.2, 7.4)	2.1 (0, 4.7)
Magnesium (mg/L)	5.8 (0, 84.9)	3.9 (1, 13.1)	1.9 (0, 10.2)
Manganese (mg/L)	0.1 (0, 3)	0 (0, 0.3)	0.1 (0, 1)
Nitrate (mg/L)	0.4 (0, 2.8)	1 (0, 21)	1 (0.3, 13.6)
Nitrogen dioxide (mg/L)	0 (0, 0.6)	0 (0, 0.1)	0 (0, 0.1)
Nitrogen/phosphate ratio	3.4 (0.1, 57.9)	8.4 (0, 202)	5.2 (1.5, 136.1)
Oxidation-reduction potential	84.8 (-184.5, 146.8)	64.5 (-188.1, 168.9)	109.6 (-150.3, 237.7)
pH	7.2 (6.3, 8.7)	6.7 (5.5, 7.6)	6.9 (5.2, 7.7)
Phosphate (mg/L)	0.1 (0, 1)	0.1 (0, 0.5)	0.2 (0, 1.1)
Sulfate (mg/L)	10 (0.2, 68.6)	5.2 (0.5, 29.2)	12.8 (1, 37.5)
Suspended solids (mg/L)	33 (8, 451)	12 (2, 64)	23 (1, 47)
Total alkalinity as CaCO_3_ (mg/l)	90 (1, 368)	34 (10, 124)	41 (12, 116)
Total dissolved solids (mg/L)	138 (37.2, 1172)	62 (22, 188)	53.9 (17.6, 221)
Total nitrogen	0.4 (0, 2.8)	1 (0, 21)	1 (0.3, 13.6)

**Fig 2:**
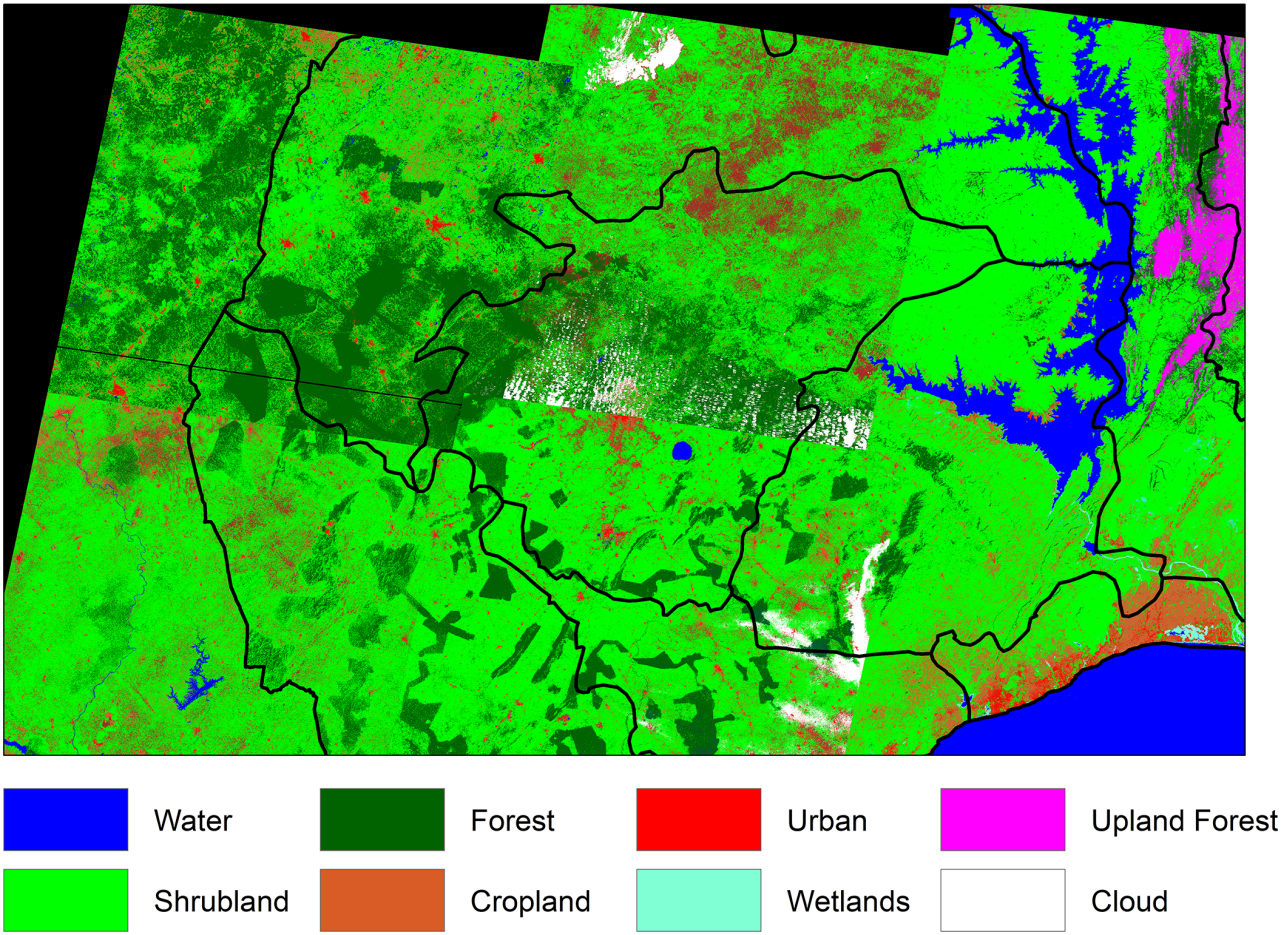
Land use/land cover in study area. Land use/land cover (LULC) from Landsat ETM+ imagery with 30 m resolution.

**Fig 3:**
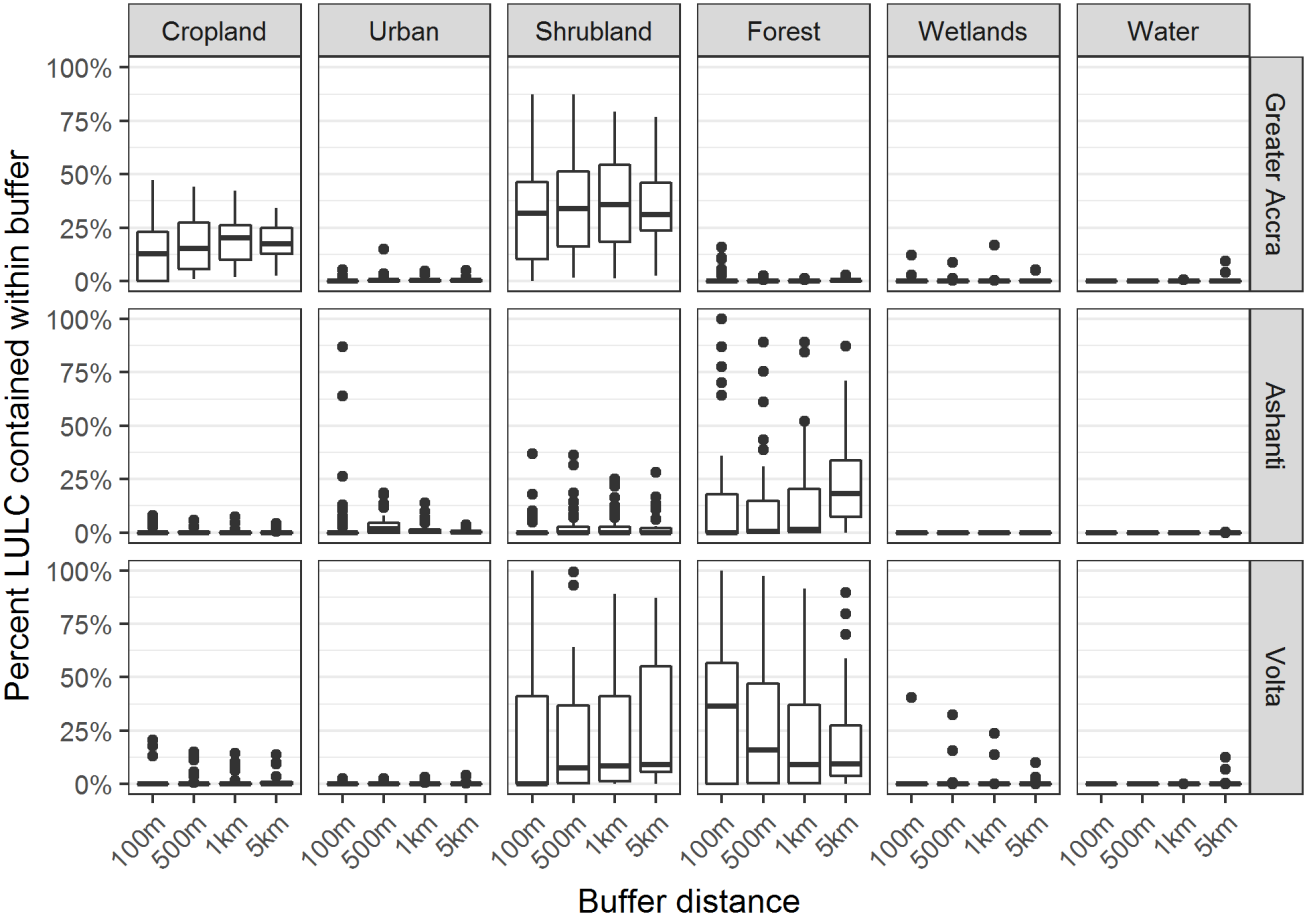
Land use/land cover distribution within buffers. Boxplots show the distribution of percent of pixels characterized by a land use / land cover type within a specified buffer distance from 29 sites in Greater Accra, 39 sites in Ashanti, and 30 sites in Volta. Plots showing only isolated dots have more than 75% of values as zero; plots showing only a horizontal bar have all values as zero.

The six predominant LULC types observed included: cropland, urban, water, forest, wetland, and shrubland (Fig 2). In the Greater Accra region most sites had cropland and shrubland present within the buffers, in the Ashanti region sites exhibited forest, and in the Volta region sites exhibited shrubland and forest (Fig 3). All three regions exhibited low percentage of urban areas within buffers, with the exception of a couple of sites in Ashanti that were characterized as mostly urban within a 100m buffer. At 30 m resolution, the percentages of water and wetlands in all buffers around the sampled sites were very low and therefore were not considered for the model selection process.

**Fig 4:**
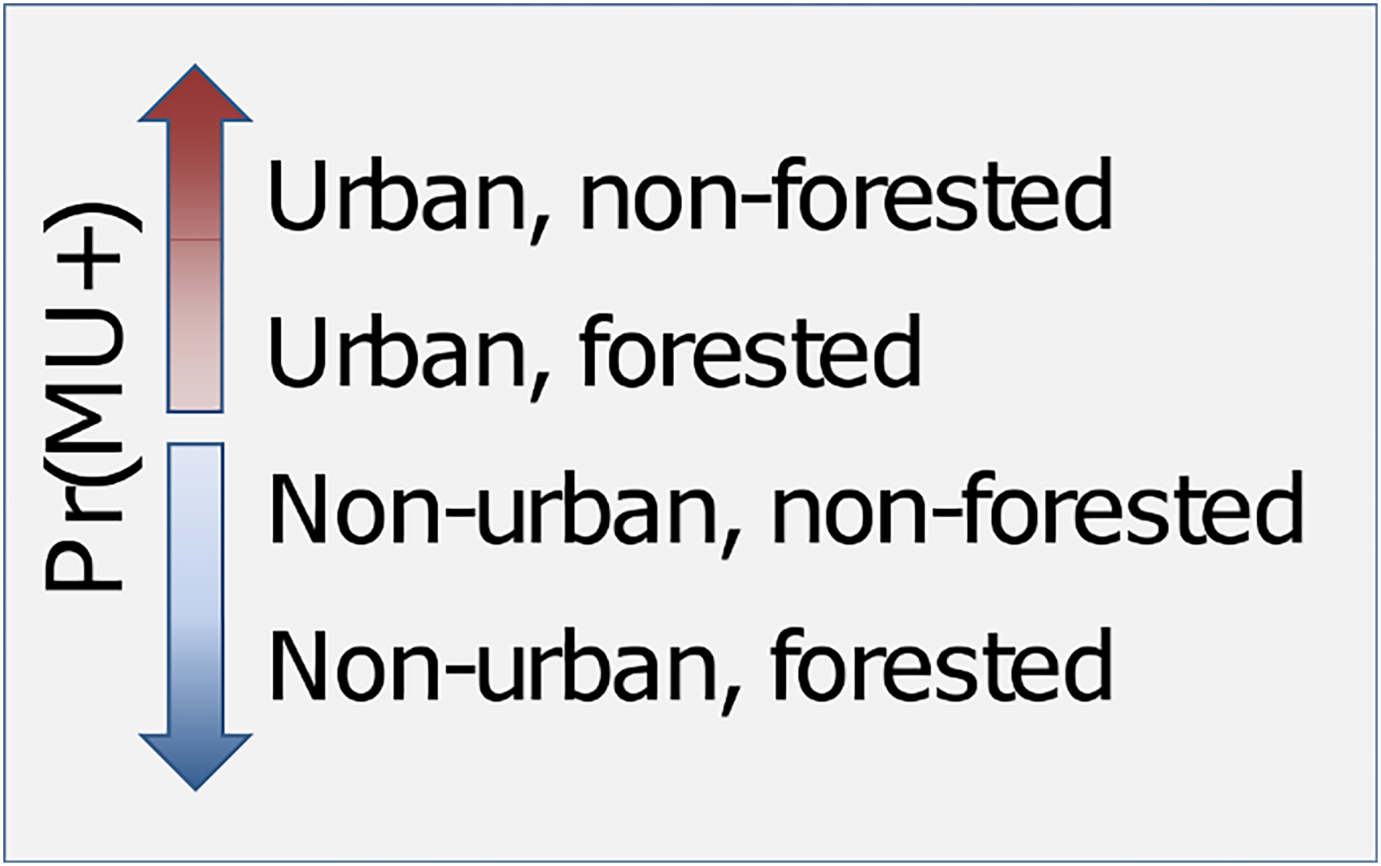
Ripley’s case-control K-function. Clustering patterns for Greater Accra were assessed up to 15 km, and for Ashanti 45 km. Shown in the solid horizontal line, the expected value of this function is 0 under the assumption of complete spatial randomness. The bold line shows the observed difference between the transformed K functions of the positive and negative sites, and the dashed lines show the theoretical confidence bounds calculated by Monte Carlo simulation.

The spatial distribution of *M*. *ulcerans* positive and negative aquatic sites was assessed for both clustering patterns and individual clusters of positive sites. The difference between the transformed *K* functions showed no significant global clustering of *M*. *ulcerans* positive sites relative to negative ones for either the Greater Accra or Ashanti regions (Fig 4). Moreover, Kulldorff’s spatial scan statistic found no significant spatial clusters of *M*. *ulcerans* positive aquatic sites.

Logistic regression identified factors associated with *M*. *ulcerans* from the distinct sets of covariates (Table 2). The AICc of the models ranged from 55.0 to 96.1, with the best fit achieved by combining covariates from all sets. This model with the lowest AICc contained seven main effects: (1) region, (2) elevation, (3) wetness index at the site, (4) standard deviation of wetness index within 500 m of the site, (5) indicator for urban land cover within 100 m of the site, (6) indicator for forest land cover within 1 km of the site, and (7) log of calcium water hardness. This model also contained three interaction terms with region: elevation, standard deviation of wetness index within 500m of the site, and calcium water hardness.

**Table 2:** Best fitting model results from five categories of variables presented in columns sorted by descending AICc, with the smallest AICc indicating the best fit.

		Water	LULC	Terrain	Landscape	All
**AICc (df)**		86.4 (6)	84.1 (4)	72.1 (7)	58.4 (9)	55.0 (11)
**Parameter estimate (SE)**						
*General*	Intercept	-2.30 (1.65)	2.15 (0.94)	-9.28 (3.75)	-19.04 (8.03)	-32.97 (14.72)
	Accra	3.28 (2.29)	-2.37 (0.84)	15.91 (4.60)	27.19 (9.53)	44.17 (17.47)
*Terrain*	Elevation			0.05 (0.02)	0.10 (0.04)	0.12 (0.05)
	Accra×Elevation			-0.05 (0.02)	-0.09 (0.04)	-0.10 (0.05)
	Wetness			-0.35 (0.16)	-0.52 (0.23)	-0.91 (0.41)
	STD(Wetness_500m_)			2.36 (1.25)	3.65 (2.13)	4.77 (3.75)
	Accra×STD(Wetness_500m_)			-5.13 (1.71)	-7.00 (2.76)	-8.67 (4.67)
*LULC*	I(Urban_100m_)		1.72 (0.88)		5.53 (2.01)	6.25 (2.47)
	I(Forest_1km_)		-1.86 (0.90)		-2.06 (1.44)	-3.01 (1.63)
*Water*	Log(CA hardness)	1.38 (0.56)				4.33 (1.97)
	Accra×Log(CA hardness)	-1.51 (0.70)				-4.41 (2.05)
	DO	-0.02 (0.01)				
	Log(NO_3_)	0.21 (0.14)				

**Fig 5:**
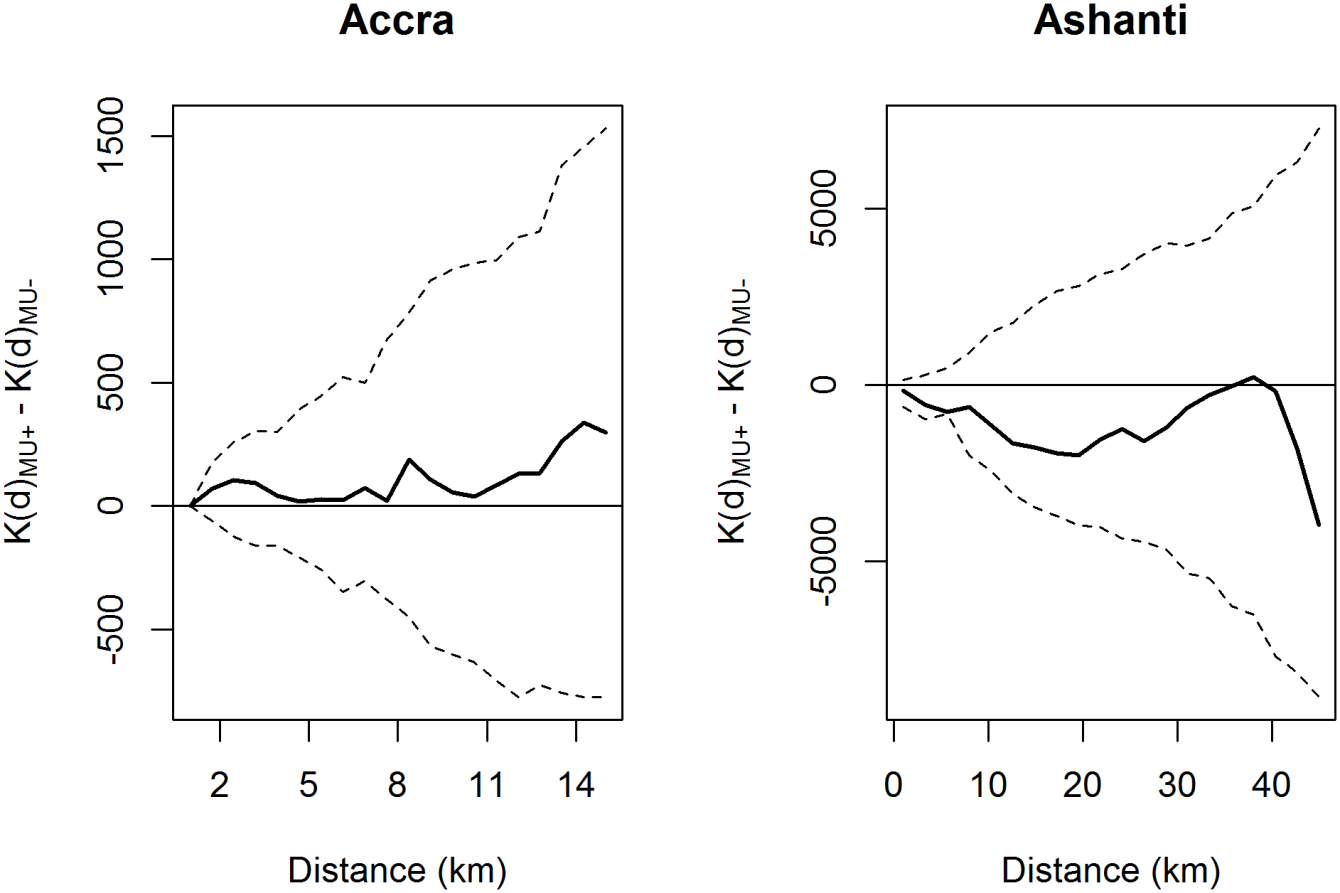
Depiction of results from best fitting model for land use / land cover variables. Land cover representing most disturbed areas are associated with a higher probability of *M*. *ulcerans* presence compared to land cover representing less disturbed areas.

The best fitting model showed that the odds of *M*. *ulcerans* presence increased as elevation increased (within the relatively modest range of elevations considered), with a more pronounced elevation effect in Ashanti than Accra. As the wetness index at the site increased, the odds of *M*. *ulcerans* presence decreased. As the standard deviation of the wetness index within 500 m of the site increased, the odds of *M*. *ulcerans* decreased in Accra but increased in Ashanti. Sites that had urban land cover present within 100 m but did not exhibit forest within 1 km had the highest odds of *M*. *ulcerans* presenc*e*. This is followed by (in order of highest to lowest) urban/forested, and then non-urban/non-forested, and lastly non-urban/forested areas had the lowest odds of *M*. *ulcerans* presence (Fig 5). *Mycobacterium ulcerans* presence was weakly negatively associated with water calcium hardness in Accra and strongly positively associated with calcium water hardness in Ashanti.

The best fitting land use/land cover, terrain, and landscape models reflected similar results to the model described above. The landscape model which included all remotely sensed covariates improved model fit when compared to the LULC or terrain model alone. The best fitting water model contained two additional variables that did not improve the fit of the final model: dissolved oxygen percent saturation (DO) and log of nitrate (NO_3_). For both Accra and Ashanti, the presence of *M*. *ulcerans* had a negative association with DO and a positive association with NO_3_.

**Fig 6:**
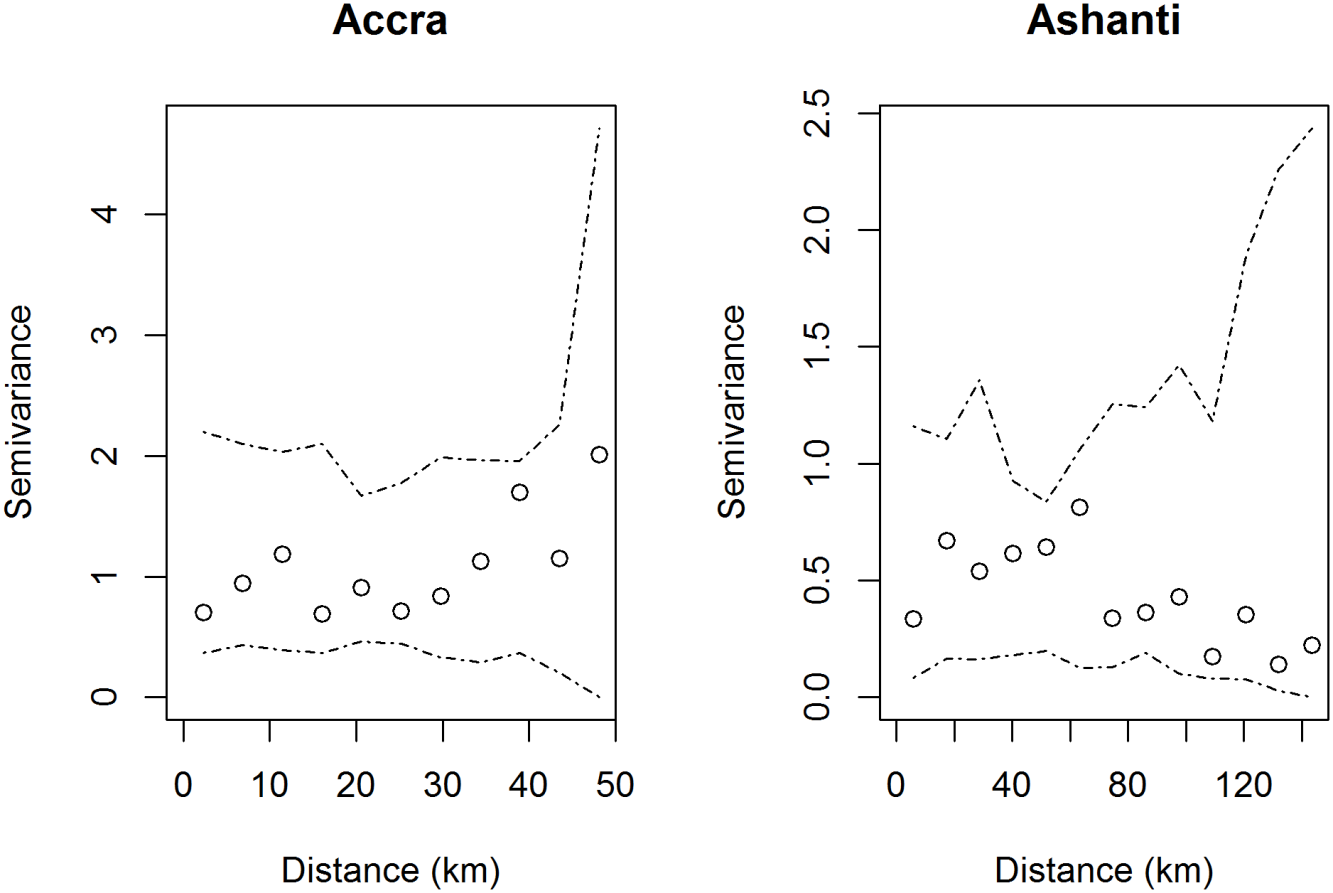
Empirical semivariogram of residuals. Circles display the observed semivariance at distance *d* and dashed lines indicate Monte Carlo simulation envelopes.

**Fig 7:**
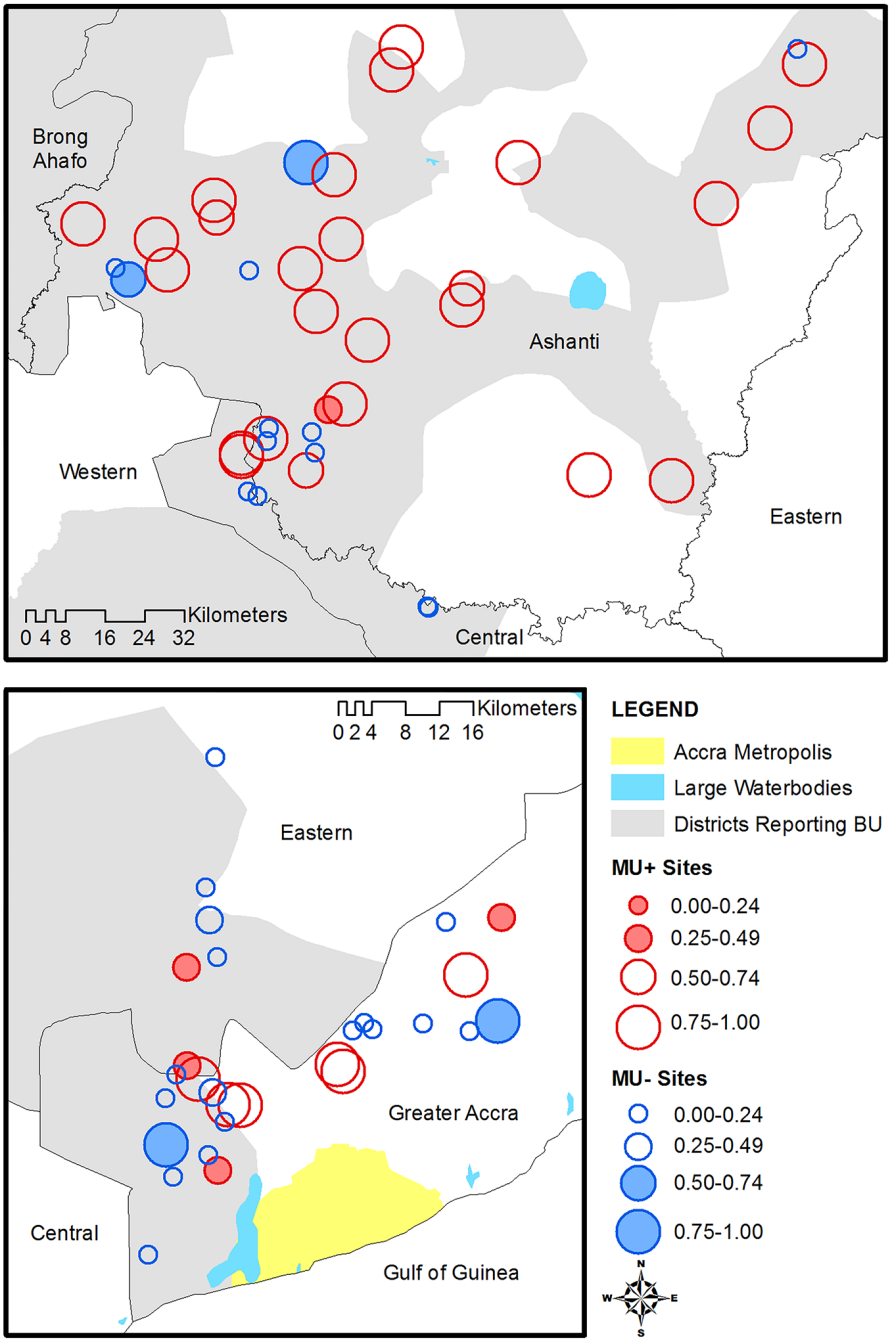
Map of the predicted probability of *M*. *ulcerans* positive based on the best fitting model. Blue circles represent sites that were actually *M*. *ulcerans* negative and red circles *M*. *ulcerans* positive. The size of the circle indicates the predicted probability of *M*. *ulcerans* positive, and shaded circles indicate poor fit (MU+ sites with low predicted probability or MU- sites with high predicted probability).

Diagnostics of the overall model best fitting model revealed no outliers in the standardized deviance residuals, and no observations with high leverage were identified. In addition, the empirical semivariogram of the residuals showed no evidence of significant spatial autocorrelation in either Greater Accra or Ashanti after adjusting for environmental covariates (Fig 6). Ashanti had lower semivariance estimates than Accra, reflecting the lower overall variance in the residuals. Three sites in Ashanti and six sites in Greater Accra indicate locations of poor model fit such that the observed outcome (*M*. *ulcerans* presence/absence) was not in accord with the predicted probability from the best fitting model (Fig 7).

Lastly, we observed variation in case reporting among the two regions. Ninety-five percent of sites in Ashanti were located within a district that reported cases, whereas 59% of the Ashanti communities actually reported cases. Sixty-nine percent of sites in Greater Accra were located within a district that reported cases, and 48% of communities actually reported cases. There was no significant association between *M*. *ulcerans* presence and district level reporting (p = 0.06) or community level reporting (p = 0.80). When added to overall best fitting model, both community level reporting and district level reporting increased AICc by approximately 3 units. Therefore, neither summary of case reporting appreciably improved model fit nor explained variation in the presence of *M*. *ulcerans* after adjusting for environmental covariates.

## Discussion

This is the first study to evaluate environmental factors associated with *M*. *ulcerans* in its natural habitat on such a broad scale. In the Greater Accra and Ashanti regions, no significant evidence of local or global clustering of aquatic sites with *M*. *ulcerans* was present, suggesting the growth of *M*. *ulcerans* may be dependent on the local environment and may exist in isolated pockets. The best fitting model of those considered included elements from both on-the-ground highly localized measurements and broad scale remotely sensed features, indicating that characteristics of local aquatic systems, general land use/land cover, and topographic features were all associated with the presence of *M*. *ulcerans*. Some of these results concur with laboratory results or speculation on *M*. *ulcerans* growth, whereas other results diverge from published literature. We explore these agreements and discrepancies for the distinct models below.

Environmental sampling from the Volta region did not detect *M*. *ulcerans*, as previously discussed by Benbow et al. (2014) [27]. Data from the Volta region were excluded from this study as the goal of this study was to investigate factors relating to variation in *M*. *ulcerans* presence. The Volta region has been historically classified as non-endemic with no reported cases of Buruli ulcer. However, recently published data of active case surveillance from the Volta region has now identified cases of Buruli ulcer [53]. It is unknown whether this is a newly emerging disease from this region, or if the lack of cases were due to a lack of reporting.

### Water variables

The best fitting model relating the presence of *M*. *ulcerans* to physical and chemical properties of water contains dissolved oxygen percent saturation, nitrate, and calcium water hardness, which concurs with other study findings. Low oxygen and increased nutrients are known indicators of eutrophic aquatic conditions that were hypothesized to be related to *M*. *ulcerans* populations dynamics [9, 32, 54], which was later confirmed through laboratory studies [23, 55].

Water hardness quantifies the mineral content in water, and is influenced naturally by the underlying geology of the system: as water passes through soil and rock it collects minerals which are deposited in the aquatic system. However, human activity on the watershed can also influence hardness. For example, drainage from mining sites can contribute a variety of minerals to an aquatic system, increasing its hardness.

The interaction effect of water hardness and region could possibly be explained by the distinct underlying geological processes in the two regions as well as by differences in human activities. Evaluation of specific water quality conditions that may enhance the presence and size of *M*. *ulcerans* populations in different regions should be studied.

Certain aquatic factors commonly discussed in the literature with *M*. *ulcerans* such as temperature and waterbody flow did not contribute to our final model. All but one of the sampled aquatic sites were below the optimal laboratory growing temperature of 30–33°C [56], suggesting environmental temperatures for population survival or growth may differ from laboratory conditions. Furthermore, BU disease occurrence has been associated with both still and moving waterbodies [1, 4, 6, 7, 10, 11, 57, 58]. However, the site classification of lentic versus lotic waterbodies did not improve model fit and therefore provided no insights into suitable aquatic conditions for *M*. *ulcerans* across the sites.

In contrast to our study comparing water quality among *M*. *ulcerans* positive and negative sites, Hagarty et al. (2015) compared water quality between three endemic and two non-endemic gold mining communities in Ghana [59]. They found that the study sites tended to be slightly acidic (pH < 7), whereas several sites assessed in our study recorded higher values of pH (pH < 8.7), especially in the Greater Accra region (S2 Fig). They also found no association between BU incidence and nutrients (nitrate, phosphate), which differed from our results in which the best fitting water model identified a positive association between nitrate and *M*. *ulcerans* presence. Lastly, as the Hagarty et al. study focused on gold mining communities, they also assessed associations between trace metals and BU incidence and found an association with arsenic, whereas we did not complete any testing on trace metals. It is possible that differences in our study results are due to differences in sampling gold mining versus non-gold mining communities; to our knowledge, none of our study sites were located in gold mining communities, though it is possible that either legal or illegal mining operations could have been located nearby of which we were unaware.

### Land use/land cover variables

We chose to consider indicators for the presence of specific LULC categories within a buffer in addition to the percentage observed for two reasons. First, the percent LULC may not have a linear relationship with the log odds of *M*. *ulcerans* presence and the true relationship may be difficult to ascertain. Second, given the short buffer distances examined combined with the relatively coarse resolution of the satellite data, indicators of LULC presence provide a more robust measure than class percentages, reducing the potential impact of unusual observations. Taken together, we find the LULC presence indicators provide additional flexibility in estimation and interpretation of observed associations within the data than the use of LULC percentages alone and provide important insight for future analyses.

We identified two fine scale (<1km) LULC variables (as indicators of presence/absence) associated with *M*. *ulcerans*. Sites with more disturbed environments (urbanized, non-forested) were more likely to have *M*. *ulcerans* present compared to less disturbed environments (forested, non-urbanized). These results are in accordance with current literature indicating disturbed environments provide conditions suitable for *M*. *ulcerans* growth by affecting the physiochemical properties of water [9, 13, 32, 57]. For example, deforestation depletes riparian cover which may increase the temperature in aquatic systems to a degree necessary for *M*. *ulcerans* growth. Furthermore, urbanization can result in increased sedimentation in aquatic systems, attenuating UV penetration, and facilitating favorable conditions for *M*. *ulcerans* growth [9, 32].

### Terrain variables

Elevation, wetness index at the site, and variability of the wetness index in the vicinity of the site were found to be associated with *M*. *ulcerans* presence. Wetness index indicates the capacity for potential water pooling based on the slope and flow direction of the DEM, with higher values indicating higher potential for pooling. Our study found a negative association between *M*. *ulcerans* presence and wetness index at the site and a positive association with elevation in the Ashanti region, which contrasted with a study of BU in Benin [15]. Areas of high wetness index or low elevation areas may be more prone to flooding or fast moving water that could wash out the natural site for *M*. *ulcerans*.

Wetness index variability had differing effects in the two regions. The positive association between wetness index variability and *M*. *ulcerans* presence in the Ashanti region could be attributed to variable wetness patterns enhancing conditions suitable for *M*. *ulcerans*.

### Overall model

The best fitting overall model contained elements from each category of covariates, which included ground-based measurements up to remotely sensed data. The residuals showed no evidence of spatial autocorrelation, indicating that a more sophisticated model taking into account the spatial locations of the sites was not necessary for our analysis. This is contrary to other studies which have shown spatial structure in Buruli ulcer case reports [17]. It is noteworthy that the semivariance of the residuals in Accra was greater than the semivariance in Ashanti, which indicated larger variability in the residuals of sites located in Greater Accra compared to Ashanti. The map of the predicted probability of *M*. *ulcerans* presence suggests more discordance between model prediction and observed outcomes in Greater Accra compared to Ashanti. Such discordant sites provide an opportunity for further investigation at specific spatial locations. *Mycobacterium ulcerans* negative sites with a high predicted probability of being positive could be re-sampled to verify the negative result, and *M*. *ulcerans* positive sites with a low predicted probability of being positive could be re-examined for unobserved covariates that may explain positive results.

### Comparing environmental associations with *M*. *ulcerans* and Buruli ulcer

While *M*. *ulcerans* is the causative agent of Buruli ulcer, it is unclear whether we should expect similarities between environmental correlates of *M*. *ulcerans* presence and those of Buruli ulcer incidence and/or prevalence at a broad scale of observation. Our best-fitting overall model measuring associations with *M*. *ulcerans* presence contains similarities to and differences from published associations between comparable landscape covariates and reported cases of Buruli ulcer, as discussed in the previous sections. In addition to differences in data quality and availability between disease surveillance and pathogen testing, simple presence of the pathogen in the environment may not be sufficient to generate measureable local increases in reported cases. For example, certain environmental factors may provide suitable habitats for *M*. *ulcerans* in addition to being collocated with high human activity areas, thus possibly increasing exposure. Conversely, other environmental conditions associated with *M*. *ulcerans* presence may not promote human interaction with the environment, thus limiting exposure to the pathogen. Moreover, some of the variables associated with BU prevalence in other settings are defined on a broader geographic scale than our variables associated with *M*. *ulcerans*. Whereas coarse spatial BU disease patterns may be identifiable on a large geographic scale due to human behavior and broad environmental characteristics, fine scale geographic characteristics are likely more relevant to understanding the local ecology of *M*. *ulcerans*.

### Associations with reported Buruli ulcer cases

We investigated whether adding the presence reported BU cases at the district or community level improved fit in our model. We did not find a significant unadjusted or adjusted association between *M*. *ulcerans* presence and either district level or community level case reporting history. However, the broader definition of district level reporting did present a marginally significant association (p = 0.06), which indicates that there may be an association between MU presence in a specific community and BU presence in a district. This gives credence to either (1) BU is not acquired where it is reported, or (2) underreporting BU cases, which was noted by Williamson et al. (2012) [20]. The lack of association between *M*. *ulcerans* presence and BU case reporting could also signify that locations of reported BU cases are not limited to locations of *M*. *ulcerans* presence, implying a more complicated connection than simple collocation and suggesting that human behavior (particularly interaction with the environment) plays a role in transmission that has yet to be defined. This highlights the need for future studies to explore further spatial relationships between human behavior and interaction with the environment, as discussed by Hausermann et al. (2012) [60].

### Limitations

Due to cost and time, sampling of each waterbody was performed only on a single day. Garchitorena et al. (2014) identified monthly variation in *M*. *ulcerans* presence among endemic sites in Cameroon [61]; such seasonal variations were not captured in this study. The study systems are highly synergistic and hypereutrophic, meaning that they are nutrient rich and often subject to periods of excessive plant and other biomass growth and decay. This results in variability in the physiochemical properties of water throughout seasons or years which we were unable to capture in order to assess how it can affect *M*. *ulcerans*. Many of the sites were riverine wetlands that experience dynamic flooding and drying periods throughout the year, which could inhibit the ability to detect *M*. *ulcerans* as such weather events could wash out natural habitats. The fluctuations in water flow resulting from heavy and sporadic rainfalls render difficult categorization of a water body as lentic or lotic at a single point in time. Moreover, temperature of the aquatic site was assessed through point measurements whereas continuous temperature measurements are preferable to accurately quantify temperature. It is likely there were fine resolution temporal changes which occurred prior to sampling at some locations that we were unable to identify. For example, lack of precipitation data at the local scale inhibited our ability to address factors (e.g., rainfall and flooding) influencing temporal changes. Moreover, the complexities of the interactions between various components of water and their effect on aquatic ecosystems were difficult to disassemble and analyze separately. Temporal studies of both BU and *M*. *ulcerans* environmental distribution are needed.

We examined remotely sensed environmental covariates in buffers at relatively short distances (<5 km) under the assumption that the presence of *M*. *ulcerans* was highly dependent on the immediate surrounding environment. Note that groundtruthing of the LULC data was not performed as part of this study due to limited resources. Moreover, the coarse resolution of the satellite imagery may have prohibited identification of small patches of land cover characteristics. For example, small bodies of water could not be identified by the satellite imagery due to the coarse resolution, and therefore this LULC class could not be statistically analyzed in relation to *M*. *ulcerans* presence. It should be noted that the coarse resolution of the DEM data could underestimate the wetness variability in buffers surrounding sites, which was measured by topographic wetness as opposed to other remote sensed imagery which measures wetness through reflectance. This study could be improved by the use of groundtruthed, high-resolution satellite imagery.

The dates of the satellite imagery (2000) did not coincide with the dates of our study time period (2005–2007) because we sought to maintain the optimum balance between satellite collection dates and quality of satellite imagery. Therefore it is possible that natural landscapes were urbanized, converted to agricultural practices, or stripped for mining during this time period which could affect our study results. Although these dates differ, important factors that may reduce this discrepancy are the multiple, unknown time lags between inoculation with MU, presentation of disease, and when individuals seek treatment. These lag times, along with the inclusion of an aggregated land cover classification, may help to reduce the impacts of this limitation.

Our results are based on the presence or absence of *M*. *ulcerans* DNA as detected by PCR from suspended material in water and plant biofilm of environmental samples. This analysis focused on *M*. *ulcerans* positive water bodies, and did not consider other potential vectors or reservoirs of *M*. *ulcerans* such as aquatic insects or mammals. In addition, the number of positive samples and the DNA abundance were not quantified. The number of *M*. *ulcerans* positive samples could possibly be underestimated due to PCR inhibitors, though previous results suggest that detection methods employed were effective in eliminating PCR inhibitors [19].

## Conclusion

The majority of findings of this study support previously posed hypotheses on the relationship between *M*. *ulcerans*, specific water conditions, and land use. Furthermore, we identified new associations between *M*. *ulcerans*, water hardness, and elevation. Our research also demonstrated complex regional interactions limiting the ability to identify a specific set of universal factors which may be indicative of high risk environments for *M*. *ulcerans*. Covariates without regional interactions could potentially be used to create maps to identify areas suitable for *M*. *ulcerans*, whereas those with regional interactions merit further investigation into the underlying cause of the interaction. Continuous remotely sensed data may be augmented by a well-planned water sampling strategy (much more time and resource intensive) to collect data for the creation of such maps. Furthermore, environmental sampling should be conducted over extended time periods (e.g., monthly for multiple years) as temporal changes in *M*. *ulcerans* and associated environmental conditions are needed to elucidate *M*. *ulcerans* ecology and BU transmission. Such temporal changes could be attributed to seasonal variations such as wet and dry season, or it could be more precisely correlated with observed weather patterns like accumulated rainfall. As it appears that *M*. *ulcerans* is present in isolated pockets in the environment, we recommend utilizing high resolution remotely sensed data in targeted areas to better quantify these associations.

In contrast to other published research in which suitable habitats corresponded directly to disease risk [62, 63], areas suitable for *M*. *ulcerans* do not necessarily correlate to areas at high risk for acquiring Buruli ulcer as human interaction with the environment likely plays an important yet undefined role in disease acquisition. Locations of reported BU cases may differ from *M*. *ulcerans* positive locations. Identifying such discordant sites where *M*. *ulcerans* is present but no BU cases are reported or areas reporting BU cases with no local presence of the pathogen could help to elucidate human behaviors associated with disease acquisition. Moreover, future studies should include temporal aspects of pathogen detection and abundance along with identified or hypothesized environmental covariates. This could help identify environmental lag times necessary to detect *M*. *ulcerans* in specific sites, much like modeling the effect of short term ambient air pollution on hospitalization due to cardiac or pulmonary disease or long term climate patterns that precede cholera outbreaks [64–66].

While few epidemiological studies have focused on the locations and environmental associations of BU disease, there have been no studies assessing the same for *M*. *ulcerans*. Knowledge of the ecology of *M*. *ulcerans* is crucial to understanding where the pathogen resides in the environment and factors which affect its growth. These details can highlight specific geographical areas in need of active disease surveillance, as well as provide insight into possible local modes of transmission. We found highly localized factors up to large-scale characterizations of environmental features were associated with the presence of *M*. *ulcerans*, and found no evidence of geographic clustering of *M*. *ulcerans* presence in neighboring aquatic systems. This research provides insights into conditions suitable for *M*. *ulcerans* growth and a basis for future research into the underlying ecology of the pathogen that causes Buruli ulcer disease.

## Supporting information

S1 Fig Water characteristics by site. Boxplots show the distribution of various physicochemical water properties from 29 sites in Greater Accra, 39 sites in Ashanti, and 30 sites in Volta.
(TIFF)

S2 Fig Water characteristics by site. Boxplots show the distribution of various physicochemical water properties from 29 sites in Greater Accra, 39 sites in Ashanti, and 30 sites in Volta.
(TIFF)

S3 Fig Water characteristics by site. Boxplots show the distribution of various physicochemical water properties from 29 sites in Greater Accra, 39 sites in Ashanti, and 30 sites in Volta.
(TIFF)
