# Supplementary figures and images for: Landscape and environmental influences on Mycobacterium ulcerans distribution among aquatic sites in Ghana
Source: PLoS One. 2017 Apr 24;12(4):e0176375. doi: 10.1371/journal.pone.0176375 (PMC5402941; doi:10.1371/journal.pone.0176375)

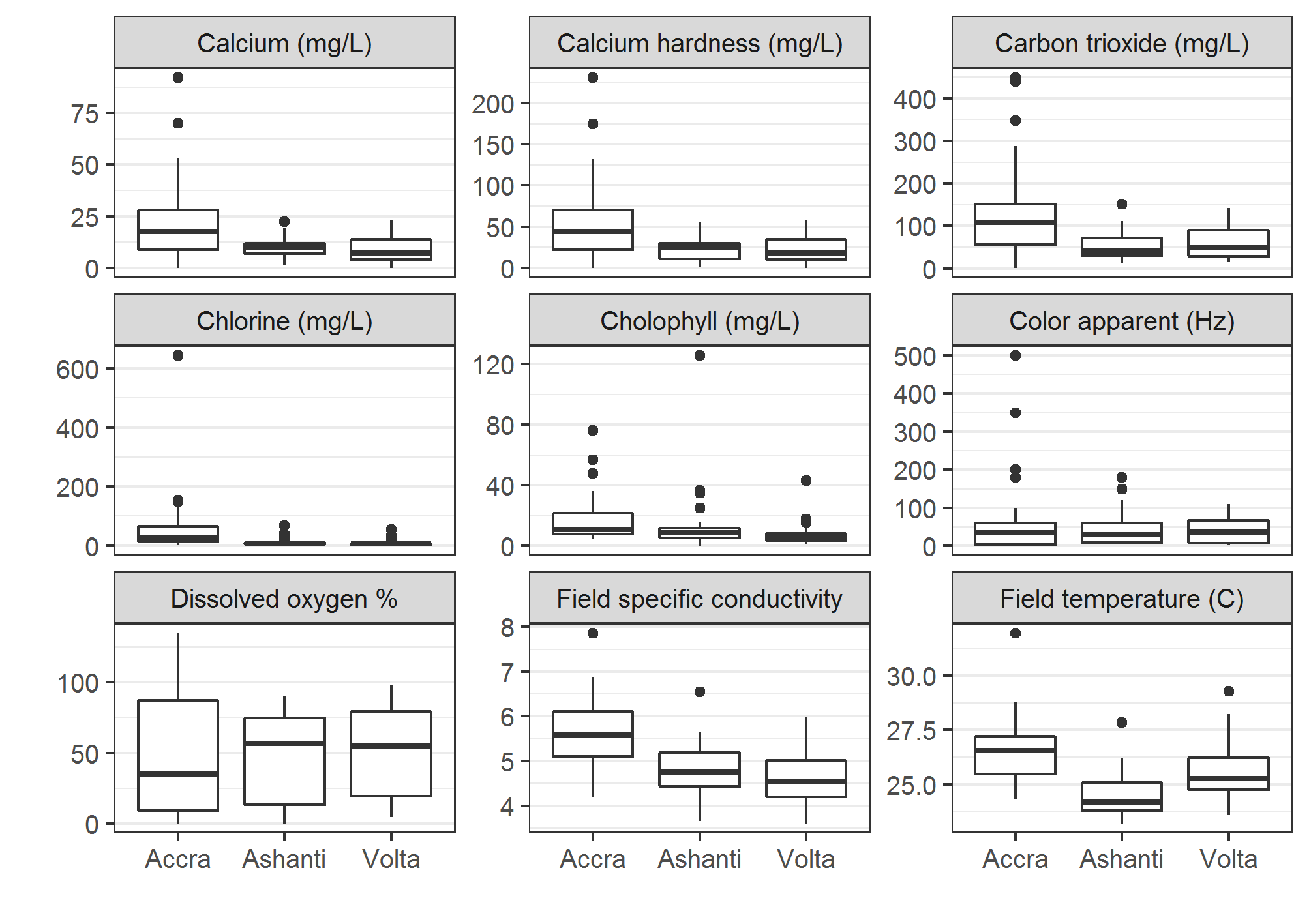

Supplement: S1 Fig — Boxplots show the distribution of various physicochemical water properties from 29 sites in Greater Accra, 39 sites in Ashanti, and 30 sites in Volta. (TIFF) [file pone.0176375.s001.tiff]

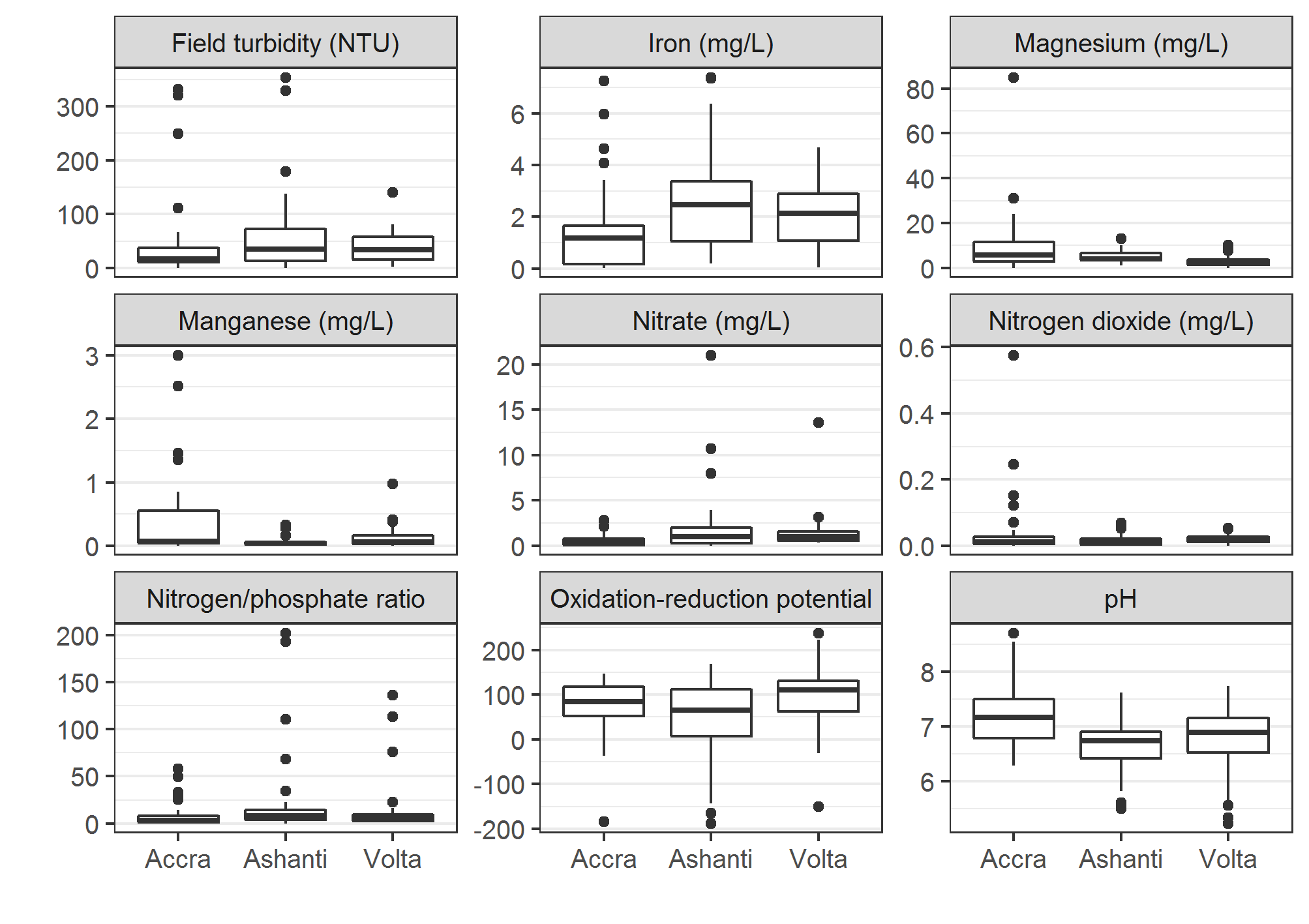

Supplement: S2 Fig — Boxplots show the distribution of various physicochemical water properties from 29 sites in Greater Accra, 39 sites in Ashanti, and 30 sites in Volta. (TIFF) [file pone.0176375.s002.tiff]

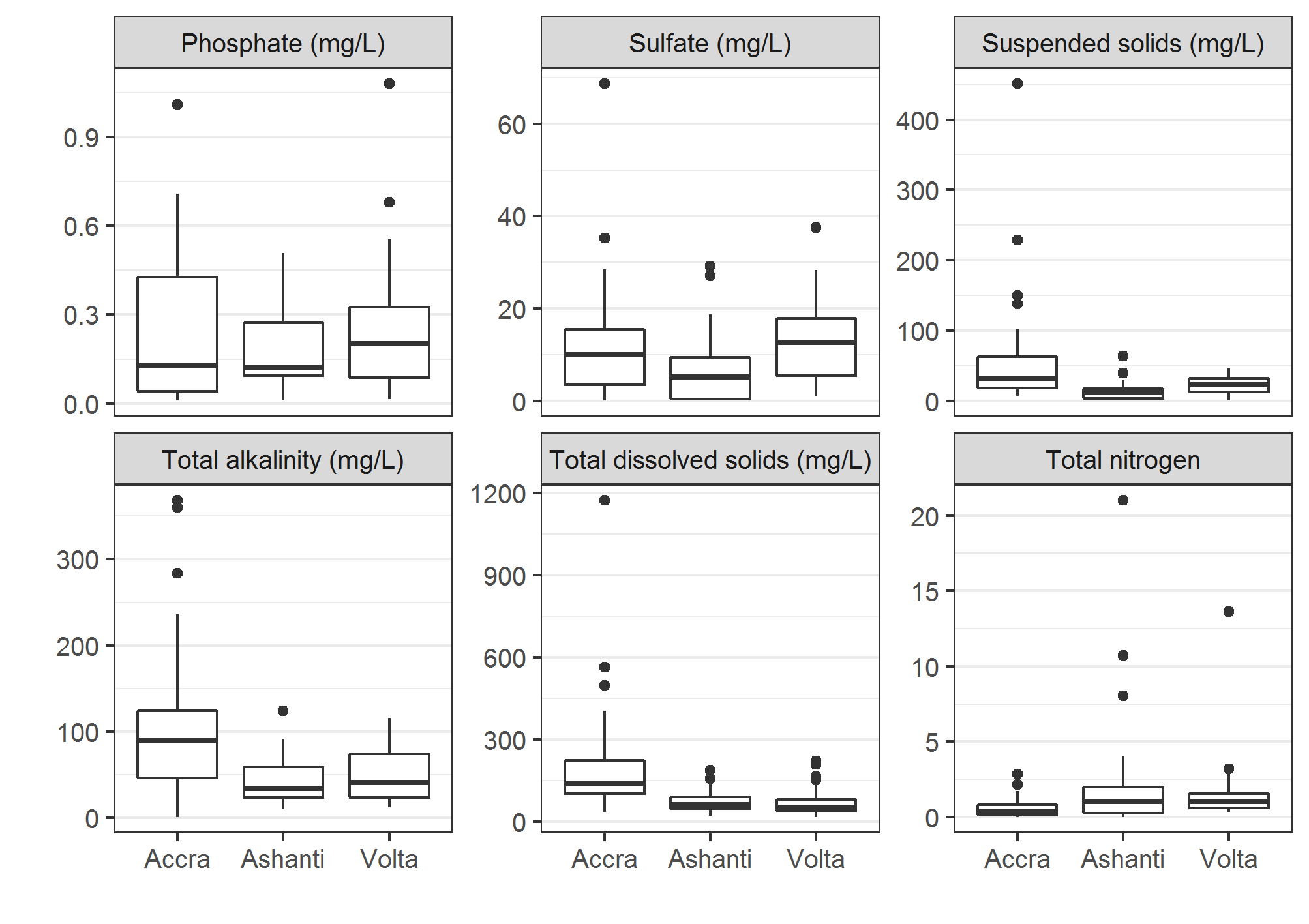

Supplement: S3 Fig — Boxplots show the distribution of various physicochemical water properties from 29 sites in Greater Accra, 39 sites in Ashanti, and 30 sites in Volta. (TIFF) [file pone.0176375.s003.tiff]
